# Utilizing machine learning to tailor radiotherapy and chemoradiotherapy for low-grade glioma patients

**DOI:** 10.1371/journal.pone.0306711

**Published:** 2024-08-20

**Authors:** Enzhao Zhu, Jiayi Wang, Weizhong Shi, Zhihao Chen, Min Zhu, Ziqin Xu, Linlin Li, Dan Shan

**Affiliations:** 1 School of Medicine, Tongji University, Shanghai, China; 2 Shanghai Hospital Development Center, Shanghai, China; 3 School of Business, East China University of Science and Technology, Shanghai, China; 4 Department of Computer Science and Technology, School of Electronics and Information Engineering, Tongji University, Shanghai, China; 5 Department of Biobehavioral Sciences, Columbia University, New York, NY, United States of America; 6 School of Medicine, National University of Ireland, Galway, Ireland; Northwestern University Feinberg School of Medicine, UNITED STATES OF AMERICA

## Abstract

**Background:**

There is ongoing uncertainty about the effectiveness of various adjuvant treatments for low-grade gliomas (LGGs). Machine learning (ML) models that predict individual treatment effects (ITE) and provide treatment recommendations could help tailor treatments to each patient’s needs.

**Objective:**

We sought to discern the individual suitability of radiotherapy (RT) or chemoradiotherapy (CRT) in LGG patients using ML models.

**Methods:**

Ten ML models, trained to infer ITE in 4,042 LGG patients, were assessed. We compared patients who followed treatment recommendations provided by the models with those who did not. To mitigate the risk of treatment selection bias, we employed inverse probability treatment weighting (IPTW).

**Results:**

The Balanced Survival Lasso-Network (BSL) model showed the most significant protective effect among all the models we tested (hazard ratio (HR): 0.52, 95% CI, 0.41–0.64; IPTW-adjusted HR: 0.58, 95% CI, 0.45–0.74; the difference in restricted mean survival time (DRMST): 9.11, 95% CI, 6.19–12.03; IPTW-adjusted DRMST: 9.17, 95% CI, 6.30–11.83). CRT presented a protective effect in the ‘recommend for CRT’ group (IPTW-adjusted HR: 0.60, 95% CI, 0.39–0.93) yet presented an adverse effect in the ‘recommend for RT’ group (IPTW-adjusted HR: 1.64, 95% CI, 1.19–2.25). Moreover, the models predict that younger patients and patients with overlapping lesions or tumors crossing the midline are better suited for CRT (HR: 0.62, 95% CI, 0.42–0.91; IPTW-adjusted HR: 0.59, 95% CI, 0.36–0.97).

**Conclusion:**

Our findings underscore the potential of the BSL model in guiding the choice of adjuvant treatment for LGGs patients, potentially improving survival time. This study emphasizes the importance of ML in customizing patient care, understanding the nuances of treatment selection, and advancing personalized medicine.

## Introduction

Gliomas constitute the bulk of primary brain tumors, accounting for 80% of all malignant brain tumors. Known for their aggressiveness, gliomas often lead to fatality within months [1, 2]. Among these, diffuse low-grade gliomas (LGGs)—including WHO grade 2 astrocytomas, oligodendrogliomas, and oligoastrocytomas [3], are the most prevalent gliomas in young adults. Characterized by their diffusive infiltration of brain parenchyma and a natural propensity to recur and progress into malignancy, LGGs pose a significant medical challenge [3].

An initial biopsy or resection is necessary for definitive diagnosis and frequently offers immediate symptom relief [1]. The standard post-operative management often includes radiotherapy (RT), which can be combined with chemotherapy (CT) [4]. However, the efficacy of these adjuvant treatments for LGG patients remains a topic of contention [4]. The RTOG 9802 clinical trial [5] recently highlighted a significant survival benefit (7.8–13.3 years) with chemoradiotherapy (CRT), establishing adjuvant CT as the standard care for LGG patients [6]. Several trials, such as RTOG 9402, EROTC 22845, and EORTC 26951, reported analogous outcomes [7, 8]. Nonetheless, contradictory evidence exists. Cairncross et al. [9], for instance, posited that CRT did not improve median survival time (MST) compared with RT alone. Similarly, Shaw et al. [10] noted that CRT enhanced progression-free survival, but it did not impact overall survival (hazards ratio (HR): 0.72, 95% CI, 0.47–1.10). Additionally, Nakamura et al. [11] suggested the lack of clear evidence supporting CRT’s benefit in treating LGGs.

These differing viewpoints suggest that the response to adjuvant therapy may vary significantly among LGG patients, given the heterogeneity in patients’ profiles, such as age, sex, and histopathologic features of tumor. The existence of such heterogeneity in treatment responses has been previously reported in LGG patients [12]. For example, EORTC 22033–26033 results suggested that younger patients (e.g., less than 40-year-old) may gain more benefits from CT [13]. Iwadate et al. [14] proposed that patients undergoing gross-total resection (GTR) might benefit from supplementary CT post-surgery. Okita et al. [15] argued that responses to adjuvant therapy could differ among LGG patients, and they stressed the necessity for a comprehensive prospective study to assess the effects of CRT on varied LGG subpopulations.

Traditionally, the efficacy of adjuvant treatments has been assessed through randomized control trials (RCTs), followed by additional subgroup analyses derived from the original RCT outcomes [16]. This can help gain a deeper understanding of how these adjuvant treatments impact various subpopulations. However, this approach encounters several challenges: 1) RCTs are costly and time-consuming; 2) RCTs often focus on average treatment effects (ATE) and overlook individual patients [17]; and 3) the lack of pre-existing clinical evidence for certain subpopulations presents a challenge for justifying the need for RCTs within these groups. Consequently, this situation often necessitates expanding the scope of RCTs to include a broader range of participants, which in turn complicates the execution of these studies. Advancements in machine learning (ML) offer a potential avenue, allowing us to infer individual treatment effect (ITE) directly from prior observational studies [18, 19].

Given the challenges in determining the optimal adjuvant therapy for LGG patients due to heterogeneity in treatment responses, our study aims to utilize machine learning models to infer individual treatment effects (ITE) and provide personalized treatment recommendations. Specifically, we seek to identify whether radiotherapy (RT) or chemoradiotherapy (CRT) is more suitable for individual patients, addressing the outlined challenges through a tailored approach.

## Methods and materials

### Study design

This study was a retrospective cohort analysis aiming to infer the ITE of adjuvant therapies. Our goal was to discern whether a patient is better suited for RT or CRT, and to identify heterogeneity in treatment responses among LGG patients. Participants for this study were selected from the Surveillance, Epidemiology, and End Results 18 (SEER) database, which comprises cancer patient data from 18 regions across the United States, thereby representing approximately 28% of the national population [20]. This study adhered to the Strengthening the Reporting of Observational Studies in Epidemiology (STROBE) reporting guidelines for observational research [21]. This study was approved by the Ethics Committee of National Cancer Institute (SEER Program). Written informed consent for participation was not required for this study in accordance with the national legislation and the institutional requirements.

This study included patients diagnosed with LGG as their primary cancer who underwent either RT or CRT from 2010 to 2015. However, several exclusion criteria were implemented. Patients falling under any of the following were excluded.

1. Age below 18;
2. Lack of clear data on tumor location, laterality, or size;
3. Ambiguity or lack of information regarding the extent of resection;
4. Unavailable survival duration data;
5. Instances of recurrent admissions.

**Fig 1:**
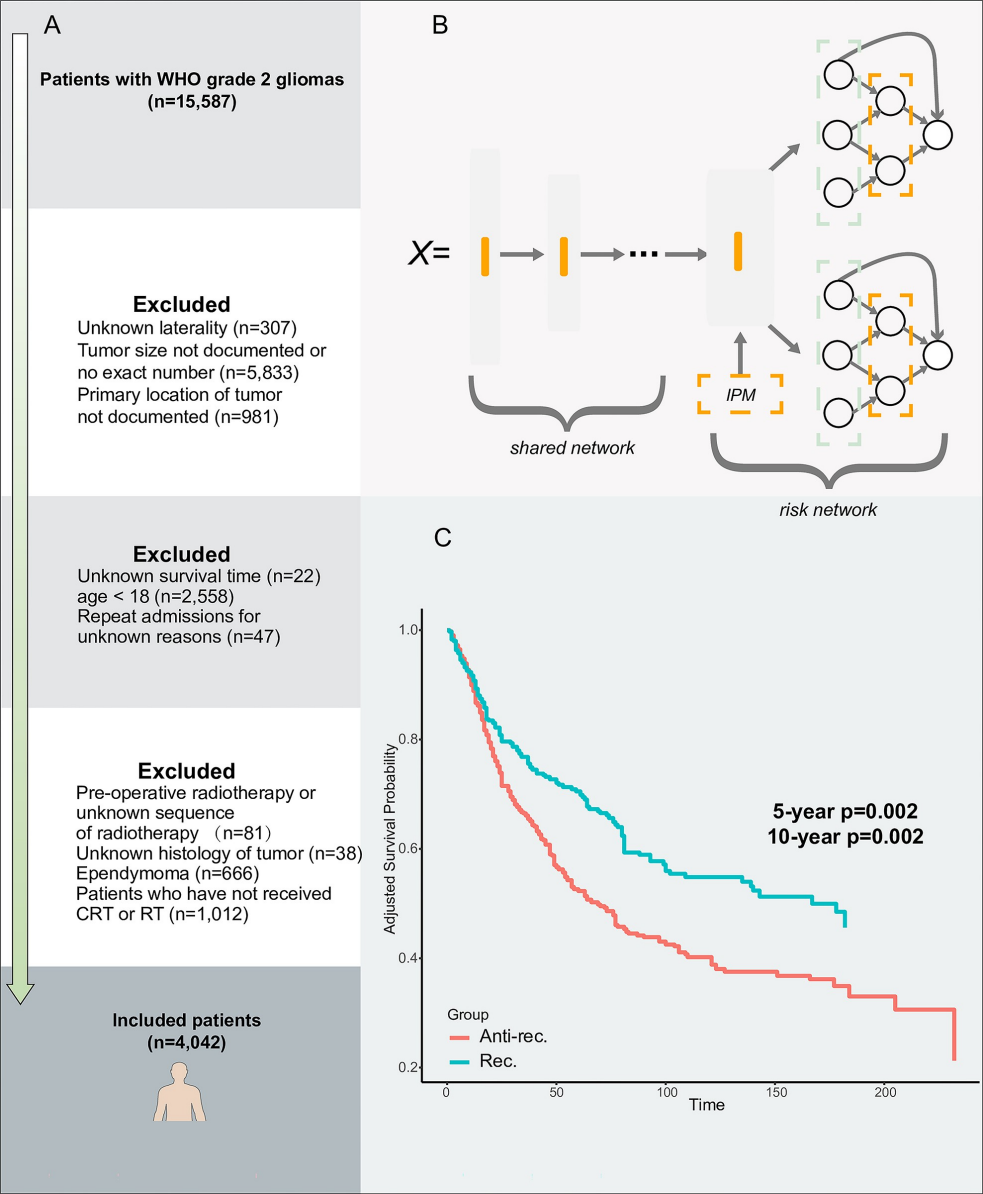
Inclusion process and model architecture. A: Detailed participant inclusion process; B: The architecture of Balanced Survival Lasso-Network; C: The inverse probability treatment weighting-adjusted Kaplan-Meier curves of the Rec. and Anti-rec. groups based on the recommendations of Balanced Survival Lasso-Network.

**Fig 1A** provides a comprehensive illustration of the participant inclusion process. Baseline data was amassed, encompassing both demographic details such as gender, age, marital status, place of residence, financial situation, and reporting states, and tumor-specific information, including histology, tumor size, location, laterality, extension, and metastasis presence. Moreover, treatment specifics, such as surgical interventions and adjuvant therapies, were included.

Surgical resections included biopsy (SEER code 20: stereotactic biopsy of brain tumor), subtotal resections (STR) (SEER code 21: Subtotal resection of tumor, lesion or mass in brain), GTR (SEER code 30: Radical, total, gross resection of tumor, lesion or mass in brain), and supra-total resections (SpTR) (SEER code 40: Partial resection of lobe of brain; SEER code 50: Gross total resection of lobe of brain), which are defined by magnetic resonance imaging as SEER required. Tumor size pertains to the measurement at the time of diagnosis, referencing the tumor’s diameter. The focal outcome under examination is brain cancer-specific survival (BCSS), a metric provided by SEER, denoting the time period between brain cancer-induced death and the initial LGG diagnosis. Categorical variables were handled using one-hot encoding during modeling.

### Machine learning algorithms and individual treatment effect

For ITE estimation, only a single factual can be observed per patient, while the counterfactual or alternative situation remains unobserved. ITE of patient *i* can be simply defined as ${ITE}_{i}={Y_{i}}^{T=1}(X_{i})-{Y_{i}}^{T=0}(X_{i})$, where *Y* represents the outcome, *T* represents a specific treatment, and *X* refers to the covariates.

Meta-learners [22], such as T-learner and X-learner, posit that intra-group differences are minimal, allowing them to separately learn the treatment effect within each group, an approach termed as the calculation of the Conditional Average Treatment Effect (CATE), which has been shown to insulate treatment recommendations from baseline differences between treatment groups [17]. In contrast, Doubly Robust Learning (DRL) [23] undertakes a two-stage estimation, utilizing covariates to predict the outcome and adjusting it with a propensity score in the first stage, and then applying another linear model to calculate CATE from outcome and treatment residuals. Causal forest (CF) [24], a distinct methodology, randomizes confounders in each decision tree branch, which enables CATE determination akin to DRL. The Representation-based [25] method employs the several approaches to balance the generating distributions across varied treatment groups. Meanwhile, Cox Mixtures with Heterogeneous Effects (CMHE) [26] operates on the assumption that the cohort consists of potential subgroups with different survival scenarios. Within each risk group, the proportional hazards assumption holds, a concept known as the conditional proportional hazards assumption. To maximize the representation of diverse risk groups, the Expectation Maximization technique is implemented.

Balanced Individual Treatment Effect for Survival data (BITES) [25] is a representation-based deep learning (DL) model. BITES contains a shared network, a Multi-layer Perceptron (MLP); and two risk networks, two MLPs, with each risk network corresponding to a distinct treatment. In shared networks, IPM is used to learn balanced representations, which has been shown to be effective for removing biased treatment administration both on the covariate space and on latent representations [27]. Risk network learns the CATE in a manner analogous to meta-learners.

Taking inspiration from the least absolute shrinkage and selection operator (LASSO), we introduced a modification to BITES, termed **B**alanced **S**urvival **L**asso-Network (BSL). In BSL, the risk network is replaced with LassoNet [28], comprising linear and non-linear components. LassoNet combines the principles of LASSO regression and neural networks, possesses inherent feature selection capabilities. It achieves this by introducing an L1 regularization term during training, which pushes the coefficients of less relevant features towards zero, effectively selecting only the most informative features. The L1 regularization acts as a regularizer, preventing the model from becoming overly complex and prone to overfitting. LassoNet only allows a feature’s non-linear participation if its penalized linear counterpart is active, thereby minimizing the influence of irrelevant features and enhancing generalizability. In contrast, standard MLPs do not inherently possess such a feature selection mechanism, often relying on external feature selection methods or using all available features. Therefore, the feature selection capability, generalization, computational efficiency, and robustness of BSL are significantly improved, which is expected to robustly predict the factual and counterfactual survival outcomes, thereby inferring more accurate ITE. The BSL inherits the overall architecture of BITES. The shared network calculates balanced (debiased) latent representation using Smoothed Optimal Transport loss [29]. Each risk network represents the corresponding treatment group, akin to T-learner. **Fig 1B** illustrates the comprehensive architecture of BSL.

### Model development and validation

This study employed ten machine learning models, namely, BSL, BITES, Cox Mixtures with Heterogeneous Effects (CMHE), DeepSurv [30], DeepSurv*, Cox proportional hazard model (CPH), random survival forest (RSF), X-learner, Doubly Robust Learning (DRL), and Causal Forest (CF). Of these, BSL, BITES, CMHE, DeepSurv, CPH, and RSF are survival regression models. Additionally, two types of ITE were defined. For survival regression models, except CMHE, which calculates ITE with RMST per the original paper [26], we defined ITE as the time difference when mortality rate reaches 50%—the Time at Risk (TaR). For other models, ITE was defined as the difference in 5-year BCSS mortality rate. In previous study [31], the raw output of the model (risk function) was used to calculate the ITE; however, in more recent study [19], TaR was recommended to be used in censored data. We therefore constructed two models with the same structure and parameters to visualize the impact of the way ITE is calculated. DeepSurv* used the raw output of the model (risk function) [31], not the TaR, to calculate ITE, meaning that treatment group-specific baseline hazards were not computed, whereas the architectures of DeepSurv and DeepSurv* were identical.

The entire patient dataset was randomly split into a training set (80% of samples) for model construction, and a testing set (20% of samples) to assess model performance and infer ITE. During training, 5-fold cross-validation was utilized for hyperparameter tuning. For each round, models were trained on four-fifths of the training set and validated on the remaining one-fifth. Training was automatically terminated if validation loss did not decrease within 1,000 iterations.

Higher ITE values indicate better BCSS outcomes and are consequently used as model recommendations. Recommendations from DeepSurv, CPH, and RSF were derived similarly to the T-learner. To study the implications of these recommendations, patients were categorized into recommended (Rec.) and anti-recommended (Anti-rec.) groups, based on the alignment of their actual treatment with model suggestions. We utilized inverse probability treatment weighting (IPTW)-adjusted hazard ratio (HR^a^), the difference in 5-year restricted mean survival time (DRMST^a^), and Kaplan-Meier (KM^a^) curves to demonstrate survival differences between the Rec. and Anti-rec. groups. These metrics, by mitigating treatment selection bias and the impact of diverse surgeries on survival, served as the fundamental performance metrics for our models.

### Model interpretation

To elucidate the decision-making process of the model, we used the odds ratio (OR) to determine the model’s recommendations based on the given covariates. Considering that patients were from different regions, we derived a mixed-effects logistic regression that predicts model recommendations from the covariates with reporting region set as random effects. The OR values thus derived could then be used to discern the key features that influence the model’s predilection to recommend RT or CRT for patients excluding potential confounders.

To facilitate more robust, time-dependent explanations of our models, we conducted SurvSHAP(t) [32], a recently proposed variation of SHapley Adaptive exPlanations (SHAP) analysis. Unlike traditional SHAP analyses that provide single-point or aggregated explanations, SurvSHAP(t) delivers explanations in the form of survival functions [33].

### Statistical analysis

All statistical analyses were performed using R version 4.1.3 and Python version 3.8. Models were built with Python packages Pytorch 2.0.0 and scikit-survival 0.19.0 with main codes were provided by original papers cited above in the Github. Metrics were calculated with R packages survival and rms. IPTW was conducted using R package ipw. The mixed-effects logistic regression was developed using R package lme4. When feeding the models, all categorical variables that contain more than three factors were processed with one-hot encoding. Continuous variables were represented as median and interquartile range (IQR), while categorical variables were denoted as numbers and percentages (%). The Kaplan-Meier (KM) curves were compared using the Log-rank test. The HR were calculated with multivariate CPH. The integrated Brier score (IBS) was calculated by sum the Brier scores over multiple time points, integrating the predictive accuracy across the entire time-to-event spectrum. Statistical significance was determined by a two-sided *P* < 0.05.

## Results

### Demographic and clinicopathological characteristics

The detailed demographic and clinicopathological characteristics of each treatment arm are delineated in **Table 1**.

**Table 1:** Baseline demographic and pathological features.

	RT (n = 1,441)	CRT (n = 2,601)
**Age, median (IQR), y**	45 (34–57)	47 (36–58)
**Tumor size, median (IQR), mm**	23 (0–50)	40 (25–58)
**Married**	831 (42.3%)	1648 (36.6%)
**Urban**	1271 (88.2%)	2281 (87.7%)
**Sex**		
** Female**	620 (43.0%)	1042 (40.1%)
** Male**	821 (57.0%)	1559 (59.9%)
**Race**		
** White**	1266 (87.9%)	2275 (87.5%)
** Other**	175 (12.1%)	326 (12.5%)
**Area of United States**		
** Midwest**	947 (65.7%)	1760 (67.7%)
** East**	208 (14.4%)	361 (13.9%)
** South**	269 (18.7%)	457 (17.6%)
** Oversea**	17 (1.2%)	23 (0.9%)
**Income**		
** Lower than $55,000**	275 (19.1%)	525 (20.2%)
** Higher than $55,000**	1166 (80.9%)	2076 (79.8%)
**Histology**		
** Astrocytoma**	663 (46.0%)	1162 (44.7%)
** Oligoastrocytoma**	498 (34.6%)	811 (31.2%)
** Oligodendroglioma**	280 (19.4%)	628 (24.1%)
**Location**		
** Frontal**	639 (44.3%)	1169 (44.9%)
** Temporal**	297 (20.6%)	569 (21.9%)
** Parietal**	184 (12.8%)	336 (12.9%)
** Occipital**	33 (2.3%)	45 (17.3%)
** Cerebellum**	24 (1.7%)	37 (1.4%)
** Cerebrum**	34 (2.6%)	106 (4.1%)
** Brainstem**	34 (2.4%)	31 (1.2%)
** Ventricle**	11 (0.8%)	23 (0.9%)
** Overlapping**	128 (12.6%)	285 (11.0%)
**Laterality**		
** Left**	418 (29.0%)	1040 (40.0%)
** Mid**	595 (41.3%)	488 (18.8%)
** Right**	428 (29.7%)	1073 (41.3%)
**Tumor extension**		
** Confined**	621 (43.1%)	1224 (47.1%)
** Ventricles**	30 (2.1%)	57 (2.2%)
** Midline**	78 (5.4%)	193 (7.4%)
**Metastasis**		
** Yes**	731 (50.7%)	1120 (43.1%)
** No/Unknown**	710 (49.3%)	1481 (56.9%)
**Extent of resection**		
** No surgey**	2 (0.1%)	12 (0.5%)
** Biopsy**	397 (26.3%)	582 (22.4%)
** Subtotal resection**	162 (11.2%)	549 (21.1%)
** Gross-total resection**	150 (10.4%)	576 (22.1%)
** Supra-total resection**	748 (51.9%)	882 (33.9%)

This study incorporated a total of 4,042 patients diagnosed with LGG. The overall BCSS rate registered at 46.5% (95% CI: 44.9%–48.0%), with a median follow-up duration of 37.0 months (IQR: 15.0–91.8 months). The median age of the participants was 46 years (IQR: 35–58 years).

Among the study population, 41.1% (1,662) were female, 61.3% (2,473) were married, and 87.6% (3,541) were white. The surgical treatments undergone by the patients varied: 23.8% (961) had biopsies, 17.6% (711) underwent STR, 18.0% (726) had GTR, and 40.3% (1,630) received SpTR. As for adjuvant treatment, 35.7% (1,441) of the patients underwent RT, while 64.3% (2,601) received CRT.

### Model performance and treatment recommendation

The performance of all models was assessed using the testing set, which incorporated 808 patients (amounting to 20.0% of the total number of patients). We measured the concordance index (C-index) and the IBS as indications of discrimination ability. Both the C-index and IBS were computed individually for the RT (C-index^RT^ and IBS^RT^) and CRT (C-index^CRT^ and IBS^CRT^) groups, given that all the models contained two separate output heads, each representing RT and CRT respectively.

More importantly, we used the HR, HR^a^, DRMST, and DRMST^a^ to gauge the degree to which BCSS improved in the Rec. group in comparison to the Anti-rec. group. These metrics were utilized as a test of the effectiveness of treatment recommendations (better prognosis following model recommendations) since they directly embody our central aim of providing individualized treatment recommendations LGG patients. For HR^a^ and DRMST^a^, we accounted for all covariates, including surgeries and adjuvant treatments, using IPTW. **S1A Fig** demonstrates the standardized mean difference (SMD) of covariates between Rec. and Anti-rec. groups. After IPTW correction, the SMD of all covariates were smaller than 0.1, which means the expected prognosis between these groups were considered balanced [34].

Detailed model performance and recommendation effectiveness are presented in **Table 2**. In the RT group, CMHE displayed the best C-index (C-index^RT^: 0.73, 95% CI, 0.70–0.75), followed by RSF (C-index^RT^: 0.70, 95% CI, 0.65–0.74), while RSF exhibited the best IBS (IBS^RT^: 0.20, 95% CI, 0.19–0.22). BSL ranked second in terms of IBS (IBS^RT^: 0.20, 95% CI, 0.18–0.23). In the CRT group, CMHE, BSL, and BITES all demonstrated the highest C-index (C-index^CRT^ of CMHE: 0.75, 95% CI, 0.73–0.77; C-index^CRT^ of BSL and BITES: 0.75, 95% CI, 0.72–0.78). CMHE achieved the best IBS (IBS^CRT^: 0.18, 95% CI, 0.17–0.19), followed by BITES (IBS^CRT^: 0.19, 95% CI, 0.17–0.21) and BSL (IBS^CRT^: 0.19, 95% CI, 0.17–0.22). Notably, CPH, although widely used, performed significantly poorly in the CRT group (C-index^CRT^: 0.58, 95% CI, 0.55–0.60; IBS^CRT^: 0.48, 95% CI, 0.44–0.53).

**Table 2:** Detailed model performance and treatment recommendation effect.

Model	HR	HR^a^	DRMST	DRMST^a^	C-index^RT^	IBS^RT^	C-index^CRT^	IBS^CRT^
BSL (n^r^ = 388)	0.52 [0.41–0.64]	0.58 [0.45–0.74]	9.11 [6.19–12.03]	9.17 [6.30–11.83]	0.67 [0.62–0.71]	0.20 [0.18–0.23]	0.75 [0.72–0.78]	0.19 [0.17–0.22]
BITES (n^r^ = 386)	0.54 [0.43–0.67]	0.58 [0.45–0.75]	8.69 [5.77–11.61]	8.78 [5.85–11.66]	0.69 [0.64–0.74]	0.23 [0.21–0.25]	0.75 [0.72–0.78]	0.19 [0.17–0.21]
CMHE (n^r^ = 406)	0.58 [0.47–0.72]	0.64 [0.50–0.82]	6.41 [3.45–9.38]	6.68 [3.56–9.06]	0.73 [0.70–0.75]	0.21 [0.19–0.22]	0.75 [0.73–0.77]	0.18 [0.17–0.19]
DeepSurv (n^r^ = 321)	0.91 [0.74–1.13]	0.71 [0.52–0.97]	3.99 [0.98–7.00]	4.07 [0.45–7.28]	0.64 [0.59–0.69]	0.32 [0.27–0.37]	0.72 [0.68–0.75]	0.41 [0.38–0.45]
DeepSurv* (n^r^ = 317)	1.05 [0.85–1.29]	1.00 [0.78–1.28]	-1.17 [-4.24–1.91]	-1.00 [-4.03–1.96]	0.64 [0.59–0.69]	0.32 [0.27–0.37]	0.72 [0.68–0.75]	0.41 [0.38–0.45]
CPH (n^r^ = 287)	0.92 [0.74–1.14]	2.51 [0.57–11.09]	2.68 [-0.40–5.76]	5.35 [2.07–8.64]	0.67 [0.65–0.74]	0.21 [0.18–0.23]	0.58 [0.55–0.60]	0.48 [0.44–0.53]
RSF (n^r^ = 412)	0.74 [0.60–0.91]	0.73 [0.58–0.93]	3.76 [0.76–6.77]	3.53 [0.30–7.06]	0.70 [0.65–0.74]	0.20 [0.19–0.22]	0.74 [0.72–0.78]	0.21 [0.19–0.23]
X-learner (n^r^ = 395)	0.71 [0.57–0.88]	0.80 [0.62–1.02]	4.01 [1.02–7.01]	4.23 [1.06–6.93]	..	..	..	..
DRL (n^r^ = 435)	0.73 [0.59–0.90]	0.73 [0.58–0.93]	4.68 [1.68–7.69]	4.73 [1.61–7.87]	..	..	..	..
CF (n^r^ = 379)	0.64 [0.52–0.80]	0.69 [0.52–0.91]	7.34 [4.40–10.27]	7.30 [4.76–10.37]	..	..	..	..

HR, multivariate hazard ratio; DRMST, the difference in restricted mean survival time; a, adjusted for all covariates using inverse probability treatment weighting; C-index; concordance index; IBS, integrated Brier score; RT, metrics calculated in radiotherapy group; CRT, metrics calculated in chemoradiotherapy group; n^r^, number of patients in the Rec. group. BSL, Balanced Survival Lasso-Network; BITES, Balanced Individual Treatment Effect for Survival data; CMHE, Cox Mixtures with Heterogeneous Effects; DeepSurv*, DeepSurv that uses risk function to calculate individual treatment effect; CPH, Cox proportional hazard model; RSF, random survival forest; DRL, Doubly Robust Learning; CF, Causal Forest.

In assessing effects, BSL demonstrated the lowest HR both before and after correction (HR: 0.52, 95% CI, 0.41–0.64; HR^a^: 0.58, 95% CI, 0.45–0.74), respectively; followed closely by BITES, with a similar HR^a^ to BSL (HR: 0.54, 95% CI, 0.43–0.67; HR^a^: 0.58, 95% CI, 0.45–0.75). CMHE ranked third (HR: 0.58, 95% CI, 0.47–0.72; HR^a^: 0.64, 95% CI, 0.50–0.82). We found a significant performance decline in DeepSurv* (HR of DeepSurv*: 1.05, 95% CI, 0.85–1.29; HR^a^ of DeepSurv*: 1.00, 95% CI, 0.78–1.28; HR of DeepSurv: 0.91, 95% CI, 0.74–1.13; HR^a^ of DeepSurv: 0.71, 95% CI, 0.52–0.97). While RSF showcased superior discrimination abilities, its recommendation performance was middling (HR: 0.74, 95% CI, 0.60–0.91; HR^a^: 0.73, 95% CI, 0.58–0.93). Among those models calculating ITE using the 5-year BCSS mortality rate, CF preformed best (HR: 0.64, 95% CI, 0.52–0.80; HR^a^: 0.69, 95% CI, 0.52–0.91). Among all the models, CPH was the worst performer overall, failing to show a positive recommendation effect (HR: 0.92, 95% CI, 0.74–1.14; HR^a^: 2.51, 95% CI, 0.57–11.09).

BSL showed consistent performance; even after IPTW correction, survival difference between the Rec. and Anti-rec. groups was increased (DRMST: 9.11, 95% CI, 6.19–12.03; DRMST^a^: 9.17, 95% CI, 6.30–11.83). BITES ranked second (DRMST: 8.69, 95% CI, 5.77–11.61; DRMST^a^: 8.78, 95% CI, 5.85–11.66). CF ranked third (DRMST: 7.34, 95% CI, 4.40–10.27; DRMST^a^: 7.30, 95% CI, 4.76–10.37), followed by CMHE (DRMST: 6.41, 95% CI, 3.45–9.38; DRMST^a^: 6.68, 95% CI, 3.56–9.06). Interestingly, post-IPTW correction, the DRMST^a^ of CPH increased, but this was not statistically significant prior to correction (DRMST: 2.68, 95% CI, -0.40–5.76; DRMST^a^: 5.35, 95% CI, 2.07–8.64). We also observed a significant performance decrease when not using baseline hazards in DeepSurv (DRMST^a^ of DeepSurv: 4.07, 95% CI, 0.45–7.28; DRMST^a^ of DeepSurv*: -1.00, 95% CI, -4.03–1.96). On the whole, excluding CF, models that calculate ITE based on mortality did not fare as well as the recently proposed survival regression models (like BSL, BITES, and CMHE), which use TaR to calculate ITE (DRMST of X-learner: 4.01, 95% CI, 1.02–7.01; DRMST^a^ of X-learner: 4.23, 95% CI, 1.06–6.93; DRMST of DRL: 4.68, 95% CI, 1.68–7.69; DRMST^a^ of DRL: 4.73, 95% CI, 1.61–7.87).

In addition, we presented the IPTW-adjusted KM curves of the Rec. and Anti-rec. groups, based on the recommendations of BSL, which showed the best recommendation effects, in **Fig 1C**. After adjusting for all baseline characteristics, surgical procedures, and adjuvant treatments in the KM curves, the results still indicated superior BCSS outcomes for the Rec. group compared to the Anti-rec. group (5-year *p* = 0.002; 10-year *p* = 0.002).

### Recognition of treatment heterogeneity

To explain the behavior of recommendations, we used a multivariate mixed-effects logistic regression that fit the recommendation of BSL from covariates in the training set, and presented the OR values in **S1 Table**. An OR value greater than 1 indicates that the presence of a feature, or an increase of one unit in this feature, makes a patient more likely to be recommended for CRT, and vice versa for RT. The SMD of covariates between RT and CRT groups before and after IPTW correction is demonstrated in **S1B Fig**. Covariates were balanced after IPTW correction.

**Fig 2:**
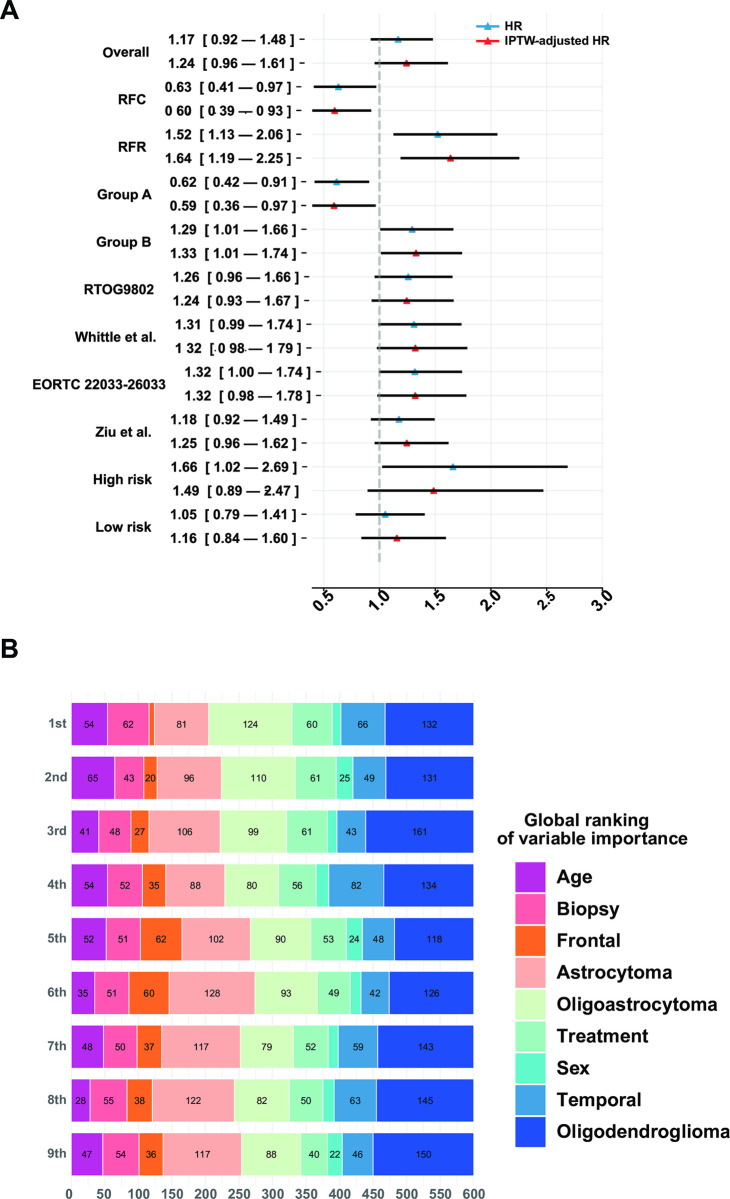
Treatment heterogeneity recognition and model interpretation. A: Treatment heterogeneity recognition; B: Model interpretation based on SurvSHAP(t). HR, hazard ratio of chemoradiotherapy; IPTW, inverse probability treatment weighting; Overall, overall population in the testing set. RFC, recommend for chemoradiotherpy group; RFR, recommended for radiotherapy group; Group A, patients under 40 with overlapping lesions or tumors crossing the midline; Group B, patients with the opposite conditions of group A. Results are compared with inclusion rules derived from the previous trials.

We presented the ATE of CRT within each subgroup of LGG patients in **Fig 2A**. The ATE were represented using HR and IPTW-adjusted HR. The CRT exhibited no statistically significant protective effect in the overall population (HR: 1.17, 95% CI, 0.92–1.48; IPTW-adjusted HR: 1.24, 95% CI, 0.96–1.61), while it presented a protective effect in the ‘recommend for CRT (RFC)’ group (HR: 0.63, 95% CI, 0.41–0.97; IPTW-adjusted HR: 0.60, 95% CI, 0.39–0.93) and became a risk effect in the ‘recommend for RT (RFR)’ group (HR: 1.52, 95% CI, 1.13–2.06; IPTW-adjusted HR: 1.64, 95% CI, 1.19–2.25). We also evaluated the protective effect of CRT in patients that meet the inclusion criteria of RTOG 9802 [35], Whittle et al. [36], EORTC 22033–26033 [37], Ziu et al. [38], high risk LGG [39], and low risk LGG [39]. However, no statistically significant results were found after IPTW correction.

Additionally, we evaluated treatment guidelines obtained by interpreting the recommendation behavior of BSL according to OR values, which indicates that CRT is more recommended for patients with younger age (OR: 0.17, 95% CI, 0.11–0.23), overlapping lesion (OR: 46.55, 95% CI, 22.45–65.18), and tumor across the midline (OR: 17.42, 95% CI, 2.56–36.29). We therefore designated the ‘recommended for CRT’ group as Group A: patients under 40 with overlapping lesions or tumors crossing the midline; while the opposite conditions are more recommended for RT (Group B). It was found that, CRT was protective in group A (HR: 0.62, 95% CI, 0.42–0.91; IPTW-adjusted HR: 0.59, 95% CI, 0.36–0.97), while it became risky in group B (HR: 1.29, 95% CI, 1.01–1.66; IPTW-adjusted HR: 1.33, 95% CI, 1.01–1.74).

### Model interpretation based on SurvSHAP(t)

We used SurvSHAP(t), the first local interpretation method introduced to date that can interpret time-to-event DL models, to interpret the functional output of BSL. For simplicity, we only considered the top eight most important variables sorted by aggregated Shapely values. We randomly sampled 600 observations from the testing set to visualize the BSL which was presented in **Fig 2B**. The horizontal bars represented the number of observations for which the importance of the variable, represented as a given color, was classified as 1st, 2nd, and so on. In "treatment", 1 represents CRT and 0 represents RT, which enters model not as a covariate but as a signal of entering different LassoNets and a usage of different treatment-specific baseline hazards.

Histology of oligodendroglioma was deemed the most important by 132 samples, followed by oligoastrocytoma which was deemed the second most important by 110 observations. The rest was astrocytoma, oligoastrocytoma, frontal location, temporal location, age, and sex. Different baseline hazards and output heads also influenced the model output significantly, which was deemed the most important and second most important by 60 and 61 observations respectively.

## Discussion

Our study reveals that the Balanced Survival Lasso-Network (BSL) model can significantly enhance the personalization of adjuvant treatment for low-grade glioma (LGG) patients. By utilizing a comprehensive dataset of 4042 LGG patients, we demonstrated that machine learning models, particularly the BSL model, can effectively discern whether an individual patient is more suitable for radiotherapy (RT) or chemoradiotherapy (CRT). This approach improves survival outcomes by providing tailored treatment recommendations. These findings underscore the critical role of individualized approaches in clinical decision-making and highlight the potential of advanced machine learning techniques in advancing personalized medicine.

The application of observational studies to infer ITE offers distinct advantages in the face of high costs and operational complexities associated with RCTs. Furthermore, determining which of the two treatments is more suitable for a specific patient is challenging in practice, since each patient is unique and has only undergone one type of treatment, making it difficult to observe and conclude in real-life scenarios. This study, therefore, utilized the principle of counterfactually predicting patient survival outcomes under different treatments through ML architectures, called counterfactual phenotyping [17]. The evolution of ML has brought about enhancements in predictive accuracy, especially with propensity score-based or representation-based methodologies ensuring unbiased estimations [17].

Interestingly, the BSL model we introduced outperformed the other models. This was indicated by the highest C-index, the lowest HR, and the largest DRMST. The BSL model leverages the feature filtering mechanism from LassoNet [28], which allows it to focus exclusively on essential potential features extracted from the MLP. Furthermore, it adopts the representation-based unbiased estimation methods from BITES [25] and can exclude confounders from observational data to learn causal associations, evidenced by the best recommendation effect with a HR^a^ of 0.58 (95% CI: 0.45–0.74), DRMST^a^ of 9.17 (95% CI: 6.30–11.83), and the 5-year *p* value of KM^a^ curves was 0.002. Following the BSL model’s treatment recommendation led to a 42% reduction in the risk of death due to brain cancer five years post-initial diagnosis, and it also extended the average 5-year BCSS time by 9.17 months. Representation-based approaches outperformed in recommendations, leading to better performance in BSL, BITES, CMHE, and CF.

Baseline hazards play a crucial role in semi-parametric survival regression models [40, 41]. She et al. [31] used the risk function of DeepSurv (without baseline hazards) to provide treatment recommendation for patients with non-small cell lung cancer, similar to DeepSurv*. However, our results suggested that baseline hazards must be taken into consideration; failing to do so could undermine the recommendation performance. In addition, the methods for calculating outcomes and ITE are areas warranting further exploration. While CMHE used RMST; DRL, X-learner, and CF employed a 5-year BCSS mortality rate to compute ITE [42]; other models utilized TaR for ITE calculation. The traditional CPH model displayed significantly weaker performance compared to the other models, raising questions about its application in decision-making for LGG patients. Therefore, it seems using TaR or RMST method has an edge over single time point outcomes, which can result in information loss and subsequently, inaccurate judgements.

Another crucial finding of our research, corroborated by existing literature, is the pronounced influence of treatment heterogeneity on treatment recommendations [41, 43]. The models were significantly impacted by the specific features of patients, such as age and tumor characteristics. CRT emerged as a more frequent recommendation for younger patients (under 40) and those with overlapping lesions or tumors crossing the midline. This aligns with prior studies that recognize younger age as a favorable prognostic factor in cancer treatments, including CRT [44–46]. Younger patients often display superior physiological resilience, rendering them more capable of enduring the intensive nature of CRT, consequently leading to improved outcomes [47]. Furthermore, in cases where patients present overlapping lesions or tumors that cross the midline, surgical removal may not be feasible due to the intricate challenges they pose [48]. Such scenarios necessitate a more targeted approach like CRT, which is capable of selectively treating the affected region while minimizing collateral damage to the surrounding healthy tissue [49]. This selective targeting is of paramount importance in brain tumors, where preserving neurological function is crucial [50, 51]. Earlier studies have affirmed the efficacy of CRT for managing such complex brain tumors [49, 52], thereby reinforcing our findings.

The study, however, comes with its own set of limitations. The retrospective design may have introduced selection bias, potentially affecting the results. This bias might arise from the inherent differences in patient characteristics and treatment protocols across different institutions. Future research should aim to mitigate such biases by employing prospective study designs and ensuring more homogeneous data collection. Additionally, advanced statistical techniques like propensity score matching or instrumental variable analysis could be utilized to address potential selection bias. Furthermore, it is essential to note that no single model should be the sole determinant for treatment decisions. Instead, a combination of several models may offer a more comprehensive assessment of patient prognosis and treatment response. Moreover, the critical role of histology in determining the model’s recommendations emphasizes the necessity for accurate histopathological diagnosis in managing LGG. Therefore, interdisciplinary cooperation between laboratory and clinical teams is of utmost importance to ensure precise characterization of LGG for optimal model functioning.

As we move forward, research should concentrate on refining these ML models and broadening their application to other types of cancer. It’s also crucial to make efforts to incorporate these models into routine clinical practice, including developing user-friendly software tools to assist clinicians in treatment decision-making.

In conclusion, our study underscores the effectiveness of ML algorithms, particularly the BSL, in predicting survival outcomes and assisting in treatment decision-making for LGG patients. Also, we observed that younger patients and those with overlapping lesions or tumors crossing the midline could derive greater benefit from CRT. The insights gained from this study hold significant implications for enhancing personalized patient care, deepening our understanding of treatment impacts, and informing future research directions in this filed. The methodologies used in these models, including their modelling [25], validation [31, 53], and interpretation [32], could offer valuable guidance for upcoming studies. The critical role of precise histopathological diagnosis is emphasized, as is the potential of incorporating these models into routine clinical practice. As personalized medicine continues to evolve, such computational models will undoubtedly become increasingly central in determining treatment approaches and improving the prognosis of patients.

## Supporting information

S1 Fig The standardized mean difference before and after inverse probability treatment weighting. (PDF)

S1 Table Interpretation of model recommendation behavior. (DOCX)

S1 File (DOCX)
