# Supplementary material for: Utilizing machine learning to tailor radiotherapy and chemoradiotherapy for low-grade glioma patients
Source: PLoS One. 2024 Aug 20;19(8):e0306711. doi: 10.1371/journal.pone.0306711 (PMC11335161; doi:10.1371/journal.pone.0306711)
Supplement: S1 Fig — (PDF) [file pone.0306711.s001.pdf]

S1 Fig. The standardized mean difference before and after inverse probability treatment weighting

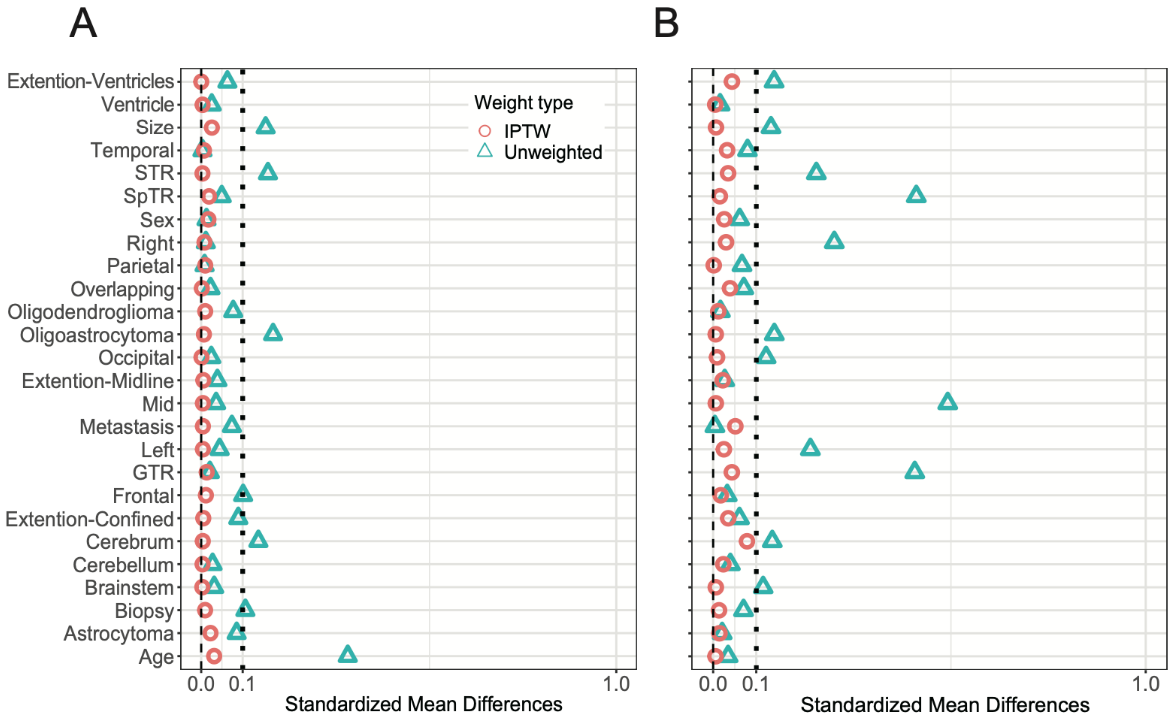

A: The standardized mean difference between Rec. and Anti-rec. groups; **B:** The standardized mean difference between radiotherapy and chemoradiotherapy groups. IPTW; inverse probability treatment weighting; STR, subtotal resection; GTR, gross total resection; SpTR, supra-total resections.
