# Supplementary material for: Utilizing machine learning to tailor radiotherapy and chemoradiotherapy for low-grade glioma patients
Source: PLoS One. 2024 Aug 20;19(8):e0306711. doi: 10.1371/journal.pone.0306711 (PMC11335161; doi:10.1371/journal.pone.0306711)
Supplement: S1 Table — (DOCX) [file pone.0306711.s002.docx]

**S1 Table.** Interpretation of model recommendation behavior.

|  | Odds ratio | 95% confidence interval |
| --- | --- | --- |
| Sex–Female | 0.05 | 0.02–0.12 |
| Age–Increase by 1 year | 0.17 | 0.11–0.23 |
| Married | 0.36 | 0.17–0.72 |
| Income–Higher than $55,000 | 8.89 | 2.94–28.78 |
| Overlapping | 46.55 | 22.45–65.18 |
| Left | 0.05 | 0.02–0.09 |
| Mid | 31.31 | 2.61–61.89 |
| Confined | 0.07 | 0.01–0.44 |
| Midline | 17.42 | 2.56–36.29 |
| Metastasis–Yes | 32.91 | 3.51–271.43 |
| Astrocytoma | 140.01 | 26.48–900.55 |
| Oligoastrocytoma | 5317.71 | 713.60–52321.10 |
| Oligodendroglioma | 31.66 | 6.01–193.72 |
| Biopsy | 0.00 | 0.00–0.54 |
| Gross-total resection | 0.00 | 0.00–0.19 |
| Tumor size–Increase by 1 mm | 1.91 | 1.72–2.15 |

The odds ratio was calculated using mixed-effects logistic regression.
