# Supplementary material for: Utilizing machine learning to tailor radiotherapy and chemoradiotherapy for low-grade glioma patients
Source: PLoS One. 2024 Aug 20;19(8):e0306711. doi: 10.1371/journal.pone.0306711 (PMC11335161; doi:10.1371/journal.pone.0306711)
Supplement: S1 File — (DOCX) [file pone.0306711.s003.docx]

**Availability of data and materials**

This study analysed public datasets that can be found here: the Surveillance, Epidemiology, and End Results Program (https://seer.cancer.gov/index.html). Dr Enzhao Zhu had full access to the analysed dataset (Grant Number: SAR0059979). According to national legislation, the individual data analysed in this study is not sharable. Data applicants should apply to the National Cancer Institute for permission to use the data program analysed in this study on their own upon scientific reasons on this website (https://seerdataaccess.cancer.gov/seer-data-access).
